# Long-Term Stability of the NIST Conical Reference Transducer

**DOI:** 10.6028/jres.116.024

**Published:** 2011-12-11

**Authors:** Steven E. Fick, Thomas M. Proctor

**Affiliations:** National Institute of Standards and Technology, Gaithersburg, MD 20899

**Keywords:** acoustic emission, long term stability, PZT, reference transducer, transfer standard

## Abstract

The National Institute of Standards and Technology (NIST) Conical Reference Transducer (CRT) is designed for purposes requiring frequency response characteristics much more uniform than those attainable with ultrasonic transducers conventionally used for acoustic emission (AE) nondestructive testing. The high performance of the CRT results from the use of design elements radically different from those of conventional transducers.

The CRT was offered for sale for 15 years (1985 to 2000). Each CRT was furnished with data which expressed, as a function of frequency, the transducer sensitivity in volts per micrometer of normal displacement on the test block. Of the 22 transducers constructed, eight were reserved for long term research and were stored undisturbed in a laboratory with well controlled temperature and humidity. In 2009, the sensitivities of these eight units were redetermined. The 2009 data have been compared with data from similar tests conducted in 1985. The results of this comparison verify the claim “Results of tests of the long term stability of CRT characteristics indicate that, if proper care is taken, tens of years of service can reasonably be expected.” made in the CRT specifications document furnished to prospective customers.

## 1. Introduction

Research at the National Bureau of Standards (NBS) addressing the design, use, and characterization of acoustic emission (AE) transducers [1–18] began in the 1970s in response to increasing needs of the nondestructive testing community. The initial outcomes of this research included the design and construction of new facilities for characterizing AE transducers, and the establishment of an AE transducer calibration service [6].

### 1.1 AE Transducer Sensitivity

For the work described in this article, AE transducer sensitivity *S* is defined to be the output voltage of the transducer per unit of dynamic displacement sensed by the transducer, where the displacement is the normal component of displacement that the surface on which the transducer is mounted would undergo if the transducer were not present [6]. Because all tests are conducted with the transducer mounted on the surface of a steel block, the sensitivity data apply only to the performance of the transducer when mounted on steel. The sensitivity is expressed in volts per μm as a function of frequency.

**Fig. 1:**
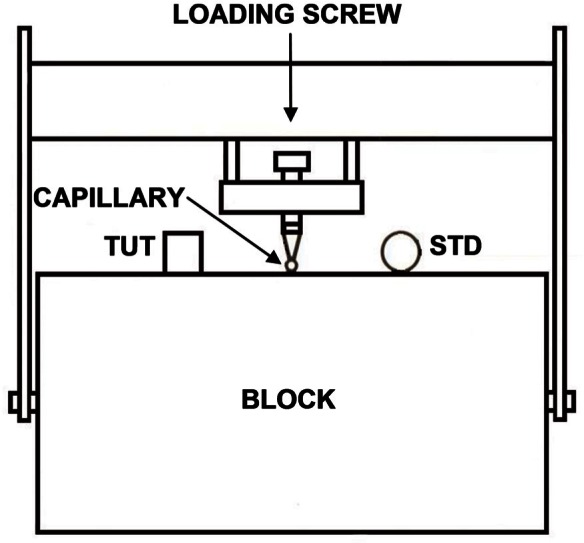
Schematic diagram of apparatus.

The steel block is a right circular cylinder 90 cm in diameter by 43 cm long. This block was specially forged in 1975. The block and its ancillary hardware are shown schematically in Fig. 1.

The transducer under test (TUT) is placed on the upper horizontal plane face of the block, which is positioned with its axis of rotation perpendicular to the floor. Elastic vibrations are induced in the block by the breaking of a glass capillary source located at the center of the circular upper surface of the block. The TUT is positioned so that its center is approximately 10 cm from the center of the circle defining the upper surface of the block. A capacitive transducer (STD), located on the upper surface of the block at a point the same distance from the source as the TUT, serves as a standard against which the TUT is compared. Because of the symmetrical locations of the STD and TUT, their displacement waveforms are expected to be identical except for the effects of the respective mechanical loadings of the block by the two transducers. The mechanical loading by the STD is insignificant. The loading effects of the TUT are taken into account by the stipulation in the definition of *S* that the displacement is that which would occur if the transducer were not present.

A two-channel digital storage oscilloscope (DSO) is used to capture voltage waveforms from the outputs of the STD and TUT during a time interval of 100 μs starting approximately 25 μs before arrival of the Rayleigh wave at both locations. Waveform data are converted to the frequency domain, and the absolute sensitivity of the TUT is calculated by comparing the responses of the TUT and STD for a set of 89 discrete frequencies between 0.1 MHz and 1 MHz.

### 1.2 Measurement Uncertainty

For the method just described, an analysis of the uncertainty of values of acoustic emission transducer sensitivity [6] was published in 1982. This analysis took into account uncertainties published in 1981 for the STD [4]. Both publications reported numerical values determined using computational procedures that became obsolete in 1992, when the procedures now used at NIST to compute uncertainty [19] were adopted. We now revisit this uncertainty analysis and recalculate the numerical results.

#### 1.2.1 Displacement Sensitivity of the STD

The displacement sensitivity of the STD can be calculated [4] from its dimensions, its distance from the block, and the applied polarizing voltage. All uncertainties associated with the STD are Type B. The combined relative standard uncertainty due to parametric measurements is 1.0 %. Because the calculations are based on a theoretical model subject to 2.0 % relative standard uncertainty, the overall uncertainty of the displacement sensitivity of the STD is estimated to be 2.2 %.

#### 1.2.2 Variability of the Capture Process

Each time a glass capillary is broken, the waveform of the vibration induced in the block will vary because of the complexity of the underlying fracture mechanics. The concomitant uncertainty can be estimated statistically from the results of repeated tests conducted sequentially, without removing the TUT from the block. This (Type A) relative standard uncertainty is estimated to be 9.0 %. The result of each test is also subject to Type B uncertainties due to the associated electronic instruments. The combined Type B relative standard uncertainty due to the instruments is estimated to be 4.3 %. The overall uncertainty due to variability of the capture process is estimated to be 10.0 %.

#### 1.2.3 Variability in the Mounting of the TUT

Each time the TUT is installed on the block, the mechanical coupling of elastic waves from the block to the TUT will vary, because the mechanics of the seating process cannot be controlled fully. The concomitant uncertainty can be estimated statistically from the results of repeated tests conducted by removing and replacing the TUT from the block for each test. The (Type A) uncertainty due to variability in the mounting of the TUT is estimated to be 10.1 %.

#### 1.2.4 Expanded Uncertainty

The combined relative standard uncertainty due to all causes just mentioned is 14.4 %. The expanded uncertainty [19], computed with coverage factor *k* = 2, is 28.8 %. For brevity hereinafter, this value is rounded and expressed as the 29 % uncertainty applicable to measured values of *S*.

## 2. Conical Reference Transducer Design

**Fig. 2:**
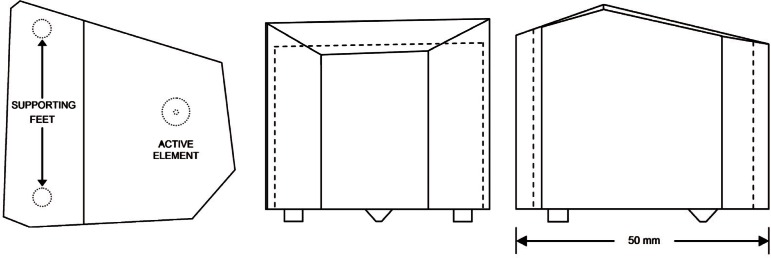
Top, front, and side views of the CRT.

The CRT comprises only two parts—the active element and the backing block. The active element is a truncated cone of axially polarized lead zirconate titanate (PZT). The backing block is a brass rhombohedron. Three orthographic schematic views are shown in Fig. 2. Unlike conventional AE transducers, the CRT has no wear plate to protect the active element from damage from contact with the surface on which the transducer is placed, and no case to protect the transducer assembly. By omitting the wear plate and case, the CRT design mitigates the complex structural resonances that can cause large variations in sensitivity with frequency.

The interface between a test object and the CRT is the electroless nickel coating on the truncated end of the active element, which is 1.5 mm in diameter. This dimension is small compared to an elastic wavelength over the 100 kHz to 1 MHz frequency range of interest, and also is small compared to the diameter of a typical conventional AE transducer. The small size reduces the aperture effect for incident off axis elastic waves [9]. The interface between the active element and the backing block is a thin layer of low temperature (60 °C) solder. The backing block has no parallel faces and no right angles. Its shape ensures that only high-order multiply reflected elastic waves can reenter the active element. The amplitude of reentrant elastic waves is minimized by the large size of the backing block. Because the active element occupies only 1 % of the area of the backing block face to which it is attached, the vast majority of reflected elastic waves cannot be intercepted by the active element. In conventional AE transducers, the active element occupies 100 % of the mating surface of the backing block, all reflected elastic waves must be intercepted by the active element, and the deleterious effects of reverberant energy can be mitigated only by the attenuation of the backing block material.

The flatness of the frequency response of the CRT could have been improved by constructing the backing block with some composite material (e.g., metal-loaded epoxy) with higher intrinsic attenuation than brass. This was not done because the long term stability of these composite materials can be compromised by phenomena not present in metals.

The CRT is furnished with a unity-gain amplifier designed to eliminate any dependence of the output of the transducer on its electrical load, and packaged in a case which forms a partial electrical shield for the transducer.

## 3. Statistical Analysis

**Fig. 3:**
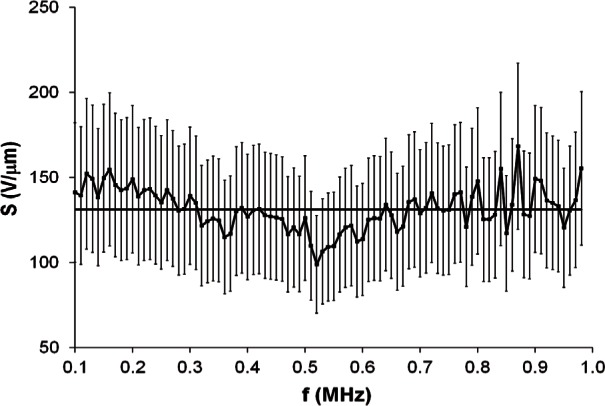
Sensitivity data from 1985 for Transducer #5. The horizontal line indicates $\bar{S}$. Error bars represent the 29 % uncertainty of measured values of *S*.

**Fig. 4:**
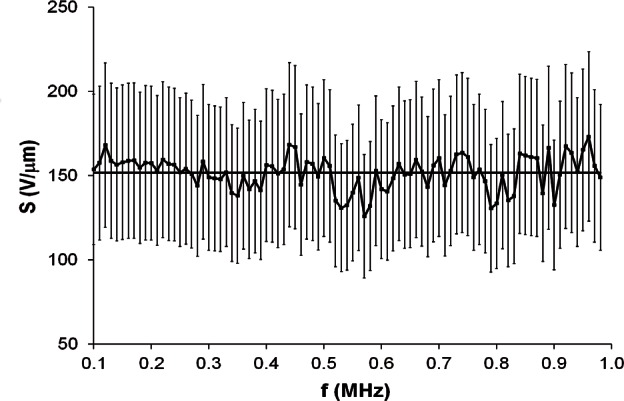
Sensitivity data from 1985 for Transducer #7. The horizontal line indicates $\bar{S}$. Error bars represent the 29 % uncertainty of measured values of *S*.

This article presents the results of a statistical analysis of the long-term stability of eight transducers after 23.8 years of storage. For each transducer, one set of sensitivity data from 1985 is available. For all transducers, the variation of sensitivity with frequency was calculated by dividing the standard deviation σ by the average $\bar{S}$, both derived from the values of *S* for all frequencies. For the eight transducers, the largest value of $\sigma /\bar{S}$ is 9.5 %, for Transducer #5. This data set is shown in Fig. 3. The horizontal line indicates $\bar{S}$. The error bars represent the 29 % uncertainty of measured values of *S*. The smallest value of $\sigma /\bar{S}$ is 6.6 %, for Transducer #7. This data set is shown in Fig. 4.

In Fig. 3, which represents the worst case for variation of sensitivity with frequency, 88 of the 89 error bars overlap the average line. In Fig. 4, which represents the best case for variation of sensitivity with frequency, all error bars overlap the average line. The observed overlap of the error bars is interpreted to suggest that it is reasonable to characterize each data set by its value of $\bar{S}$.

Table 1 presents values of $\bar{S}$ and $\sigma /\bar{S}$ of the sensitivities determined in 1985 for all frequencies, and the standard deviation σ expressed as a percentage of $\bar{S}$.

The fact that all values of $\sigma /\bar{S}$ are much smaller than the 29 % uncertainty is interpreted to confirm that it is reasonable to characterize every data set by the average value of its sensitivities for 89 frequencies.

For the eight transducers, the range of the tabulated values of $\bar{S}$ is 30 V/μm. Half of this range is equivalent to 10.2 % of the average of the values of $\bar{S}$ for all transducers, and is well within the 29 % estimated uncertainty. This is taken to confirm that in 1985, less than a year after having been constructed, the eight transducers were nominally identical.

**Fig. 5:**
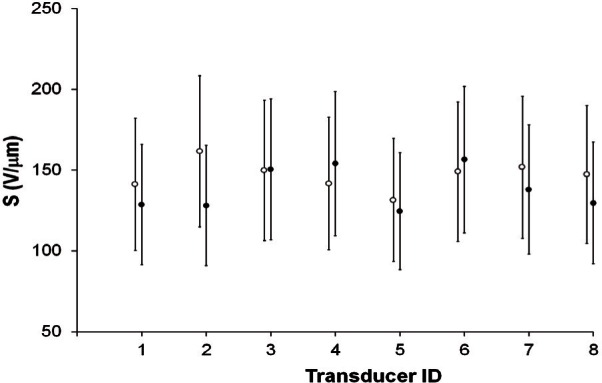
Average sensitivities from 1985 and 2009 for all transducers. Hollow circles show values of $\bar{S}$ from 1985; solid circles depict grand average sensitivities for 2009. Error bars show estimated uncertainties.

Each transducer was tested at least six times in 2009. For each transducer, average values of $\bar{S}$ for each test were calculated. Simple averaging of these results was used to determine the grand average sensitivity for the tests conducted in 2009. The results are shown in Fig. 5, which also shows the values of $\bar{S}$ from 1985, plotted for clarity as hollow circles slightly to the left of the solid circles that represent the grand average sensitivity for 2009. The error bars reflect the estimated uncertainty. For each transducer, the average of one data set falls within the error limits of the other, suggesting that the change in average sensitivities from 2009 and 1985 is within the limits of the estimated uncertainty.

For each transducer, $\sigma /\bar{S}$ was calculated for each test. Simple averaging of these results was used to determine grand average values of the variation of sensitivity with frequency for the tests conducted in 2009. For further analysis, we define the parameter *R* to be the ratio calculated by dividing the grand average sensitivity for the tests conducted in 2009 by the single value of $\bar{S}$ determined in 1985. The value of the associated parameter *R*-1 would be zero if there had been no change in sensitivity between 1985 and 2009.

Table 2 presents for each transducer the grand average values of $\bar{S}$ and $\sigma /\bar{S}$, and the value of *R*-1.

The fact that all grand average values of $\sigma /\bar{S}$ are significantly smaller than the 29 % estimated uncertainty is interpreted to confirm that it is reasonable to characterize every data set by the average value of its sensitivities for 89 frequencies.

For the eight transducers, the range of the tabulated values of $\bar{S}$ is 32 V/μm. Half of this range, 16 V/μm, is equivalent to 11.5 % of the average of the values of $\bar{S}$ for all transducers, and is well within the 29 % estimated uncertainty. This is taken to confirm that in 2009, some 24 years after having been constructed, the eight transducers were nominally identical.

The range of the tabulated values of *R*-1 is 30 %. Half of this range, 15 %, is well within the 29 % estimated uncertainty. The worst case value of *R*-1 is −21 %, which is also well within the estimated uncertainty. These results are interpreted to confirm that, for every transducer, the change in average sensitivity between 1985 and 2009 is within the limits of the estimated uncertainty.

## 4. Conclusion

A set of eight NIST CRTs constructed and characterized in 1985 has been stored undisturbed under well controlled temperature and humidity. In 2009, the sensitivity of each CRT was redetermined at least six times. Analysis and comparison of the data sets from 1985 and 2009 indicates that for all eight CRTs, the change in sensitivity did not exceed the 29 % estimated uncertainty applicable to any value of sensitivity. For the worst transducer, the change in sensitivity was −21 % over 23.8 years. This result determines the estimated worst-case drift rate, −0.87 %/year, for the CRT design. At this worst-case rate, accumulated monotonic drift would equal the estimated uncertainty after 33.1 years. This result is consistent with the preliminary finding “Results of tests of the long term stability of CRT characteristics indicate that, if proper care is taken, tens of years of service can reasonably be expected.” reported in the CRT specifications document furnished to prospective customers.

## Figures and Tables

**Table 1:** Transducer sensitivity statistics for data from 1985

Transducer	$\bar{S}$ (V / μm)	$\sigma /\bar{S}$ (%)
#1	141.1	9.1
#2	161.5	6.8
#3	149.8	7.2
#4	141.7	7.2
#5	131.5	9.5
#6	149.1	7.3
#7	151.7	6.6
#8	147.2	6.9

**Table 2:** Transducer sensitivity statistics for data from 2009

Transducer	gr. avg. $\bar{S}$ (V / μm)	gr. avg. $\sigma /\bar{S}$ (%)	*R*-1 (%)
#1	128.6	13	−9
#2	128.1	11	−21
#3	150.4	10	0
#4	154.0	9	9
#5	124.6	11	−5
#6	156.5	17	5
#7	137.9	15	−9
#8	129.6	12	−12
